# *mcp*,* aer*,* cheB,* and *cheV* contribute to the regulation of *Vibrio alginolyticus* (ND‐01) adhesion under gradients of environmental factors

**DOI:** 10.1002/mbo3.517

**Published:** 2017-07-25

**Authors:** Lixing Huang, Lu Wang, Xiangzhi Lin, Yongquan Su, Yingxue Qin, Wendi Kong, Lingmin Zhao, Xiaojin Xu, Qingpi Yan

**Affiliations:** ^1^ Fisheries College Key Laboratory of Healthy Mariculture for the East China Sea Ministry of Agriculture Jimei University Xiamen Fujian China; ^2^ Third Institute of Oceanography State Oceanic Administration Xiamen Fujian China; ^3^ College of Ocean & Earth Sciences Xiamen University Xiamen Fujian China

**Keywords:** adhesion, environmental stresses, *Vibrio alginolyticus*

## Abstract

Adhesion is a key virulence factor of pathogens and can be affected by the environment. Our previously research with RNA‐seq indicated that *mcp*,* aer*,* cheB,* and *cheV* might play roles in the regulation of adhesion in *Vibrio alginolyticus* (ND‐01). In order to determine whether and how environmental factors affect adhesion through these genes, gene silencing was performed followed by quantitative real‐time PCR (qRT‐PCR), RNAi, transmission electron microscopy, and adhesion, capillary, and motility assays to verify how these genes influence adhesion. Silencing these genes led to deficiencies in adhesion, chemotaxis, flagellar assembly, and motility. The expression levels of *cheA*,* cheW*, and *cheY*, which are important genes closely related to the functions of *mcp*,* aer*,* cheV*, and *cheB*, were significantly downregulated in all of the RNAi groups. The expression of *mcp*,* aer*,* cheV*, and *cheB* under different gradients of temperature, pH, and salinity and after starvation for various durations was also detected, which showed that these genes were sensitive to certain environmental stresses, particularly pH and starvation. Our results indicated that *mcp*,* aer*,* cheB,* and *cheV*: (1) are necessary for ND‐01 adhesion; (2) play key roles in the bacterial chemotaxis pathway by controlling the expression of downstream genes; (3) might affect adhesion by impacting motility, though motility is not the only route through which adhesion is affected; and (4) contribute to the regulation of ND‐01 adhesion in natural environments with different temperatures, pH levels, and salinities as well as after various starvation periods.

## 1 INTRODUCTION

The bacterial chemotaxis pathway is composed of a receptor module that senses gradients in their chemical environment and generates the network activity and an adaptation module that allows the bacteria to maintain a steady‐state activity in spite of ambient conditions (Yuan & Berg, 2012). The relation between chemotaxis and adhesion has been extensively studied in eukaryote, such as the lymphocyte and the cancer cells (Reddy & Kalraiya, 2006; Wendt et al., 2016), whereas the study about prokaryotes has also been performed on some critical pathogens although not become widespread. For example, chemotaxis of cholera vibrios is proved to facilitate the association of these bacteria with the mucosal surface (Freter, O'Brien, & Halstead, 1978); chemotaxis of *Vibrio furnissii* is proved to catalyzes the first step in colonizing chitin (Bassler, Gibbons, Yu, & Roseman, 1991); chemotaxis was also proved to play key roles in the colonization of adherent‐invasive *Escherichia coli*. Therefore, more and more evidences have shown that chemotaxis pathway might play key roles in the adhesion of various pathogens, but the way in which the genes involved in the bacterial chemotaxis pathway affect adhesion might be different among pathogens (Olsen et al., 2013).

*Vibrio alginolyticus*, a gram‐negative halophilic bacterium, is a normal inhabitant of coastal and estuarine environments in warm tropical regions and also represents one of the leading opportunistic pathogens in marine animals, including grouper, large yellow croaker, sea bream, Kuruma prawn, abalone, and carpet shell clam (Gu et al., 2016). *V. alginolyticus* represents a sustained threat to aquaculture industries and ecosystems around the world (Ruwandeepika et al., 2012). *V. alginolyticus* could also cause superficial wound infections and other intra/extraintestinal diseases in humans (Austin 2010; Uh et al., 2001).

During the pathogenic process of bacteria, attachment to external surfaces of a host is an important initial step in the colonization and subsequent occurrence of infection (Tsilia, Kerckhof, Rajkovic, Heyndrickx, & Van de Wiele, 2015). Indeed, without adhesion to its host, *V. alginolyticus* might not be able to persist at the initial infection focus, which would result in the absence of colonization and disease (Balebona et al., 1998). Although bacterial adherence is one of the most active areas of study in the field of infectious diseases (Tsilia et al., 2015), the molecular mechanisms of *V. alginolyticus* adhesion have not been widely investigated.

Because RNA‐Seq can survey the entire transcriptome in a very high‐throughput and quantitative manner (Yang, Zheng, Cui, Yang, & Chen, 2016), allowing an assessment of the complexity of the transcriptome and facilitating the identification of genes (Gao et al., 2015), this technology has been widely applied to uncover genes and pathways in microbes. In our previous study, we found that treatment with stresses (including Cu, Pb, Hg, and low pH) could reduce the adhesion of *V. alginolyticus* (ND‐01). To better understand the mechanism(s) underlying the regulation of adhesion, the adhesion deficient strains were analyzed using RNA‐Seq. The results showed that these stresses can markedly influence the expression of *mcp*,* aer*,* cheB,* and *cheV* (Kong et al., 2015).

*mcp* encodes a methyl‐accepting chemotaxis protein that senses chemical gradients (Parales, Luu, Hughes, & Ditty, 2015), and *aer* encodes an aerotaxis, energy (Bibikov, Barnes, Gitin, & Parkinson, 2000; Samanta, Widom, Borbat, Freed, & Crane, 2016), and redox sensor. *cheV* and *cheB* encode a methyltransferase and methylesterase responsible for the methylation and demethylation of methyl accepting chemo receptors (MCPs), respectively (Cho, Crane, & Park, 2011). So far, relatively few studies have been conducted on the relationship between these genes and the bacterial adhesion. For example, the fraction of *Marinobacter adhaerens* HP15 attaching to the diatom surface is significantly decreased in *cheA*,* cheB*,* chpA*, and *chpB* mutants compared with wild type (Sonnenschein, Syit, Grossart, & Ullrich, 2012). In *Salmonella Typhimurium*,* cheB*, but not *cheA*, is essential for adhesion (Olsen et al., 2013), whereas *cheB* is not necessary for adhesion in *S. Dublin* (Olsen et al., 2013). The *S. Typhimurium cheB* mutant was significantly reduced in adhesion by 84.4%. Moreover, the deletion of *mcp* affects important phenotypes, such as motility, biofilm formation, and colonization (Chandrashekhar et al., 2015), and the swarming and immobilization of *Pseudomonas aeruginosa* along wounds is abolished in strain PAO1 (PAO1ΔcheYZABW, which does not express *cheY*,* cheZ*,* cheA*,* cheB,* or *cheW*) (Schwarzer, Fischer, & Machen, 2016) Therefore, some evidence have shown that these genes might be important for the adhesion of several pathogens. Regardless, the mechanism through which they affect adhesion might differ among pathogens (Olsen et al., 2013). According to RNA‐Seq results, *mcp*,* aer*,* cheB,* and *cheV* might exert a marked influence on the adhesion process of ND‐01, and the sensitivity of these genes to environmental stresses might constitute a mechanism through which environmental conditions affect adhesion. Studies of the underlying mechanisms and the effects of environmental factors are of great importance and might help in the development of measures to prevent infection.

The aims this study were to determine (1) whether *mcp*,* aer*,* cheB,* and *cheV* are involved in the regulation of ND‐01 adhesion and (2) the mechanism through which these genes affect adhesion under gradients of environmental factors.

## 2 MATERIALS AND METHODS

### 2.1 Bacterial strain and culture conditions

Pathogenic *V. alginolyticus* (ND‐01) was isolated from naturally infected large yellow croakers by our laboratory (Kong et al., 2015). ND‐01 was grown in Luria–Bertani (LB) broth supplemented with 2% NaCl with shaking (220 rpm.).

The effects of different temperatures were investigated as described by Huang et al. (2016). ND‐01 was cultured overnight in LB broth (supplemented with 2% NaCl, pH7) at 4°C, 15°C, 28°C, 37°C, and 44°C. Six replicates were included for each treatment. The bacteria were then harvested and resuspended, and the bacterial suspensions were equilibrated at the same temperature for 30 min. The cells were used for RNA extraction.

The effects of various pH values were assessed as described by Huang et al. (2016). ND‐01 was cultured at 28°C overnight in LB broth (supplemented with 2% NaCl) at different pH values (pH5, 6, 7, 8, and 9), adjusted using HCl and NaOH. The bacterial cultures were washed with phosphate‐buffered saline (PBS) (pH 5, 6, 7, 8, and 9) (Yan, Chen, Ma, Zhuang, & Wang, 2007) and adjusted to an OD_560_ of 0.3. RNA extraction and qRT‐PCR were then performed. Six replicates were performed for each treatment.

The influences of different salinities were evaluated as described by Huang et al. (2016). ND‐01 was cultured at 28°C overnight in LB broth at different salinities (0.8%, 1.5%, 2.5%, 3.5%, and 4.5%), and six replicates were included for each treatment. The bacterial cultures were washed using PBS at different salinities (0.8%, 1.5%, 2.5%, 3.5%, and 4.5%) (Yan et al., 2007) and adjusted to an OD_560_ of 0.3. The bacterial suspensions were then subjected to RNA extraction and qRT‐PCR.

The influence of starvation was evaluated as described by Huang et al. (2016). ND‐01 was suspended in PBS, and the bacterial suspensions were adjusted to an OD_560_ of 0.3, starved at 28°C for 1, 3, 5, and 7 days, and then sampled for RNA extraction and qRT‐PCR. Six replicates were included for each treatment. Culturable ND‐01 cells were counted using plate counting (PC) as described by Luo et al. (2016).

*E. coli* strain SM10 was purchased from TransGen Biotech (Beijing, China) and cultured in LB broth (220 rpm.) or on LB plates at 37°C.

### 2.2 Transient gene silencing

Short interfering RNA (siRNA) was designed and synthesized according to gene sequences by GenePharma Co., Ltd. (Shanghai, China). siRNA sequences and the negative control are listed in Table S1.

Electroporation was performed using a Bio‐Rad MicroPulser (Bio‐Rad Laboratories, Inc.) as described by Wang et al. (2015). A 100‐μl volume of competent cells was mixed with 2 μl siRNA (20 μmol/L). After storing on ice for 30 min, the cells and siRNA were transferred to a cuvette. Immediately following electroporation (1.8 KV, 6 ms), 900 μl LB medium was added, and the mixture was then incubated at 28°C for 1, 3, 6, 9, and 12 hr prior to RNA extraction and RT‐PCR.

ND‐01 subjected to RNAi treatments displayed significant silencing at 1–6 hr (mentioned below). The cells were recovered at 28°C and 50 rpm. for 2 hr after electroporation to conduct the in vitro adhesion assays described below.

### 2.3 Stable gene silencing

Stable gene silencing was performed according to the study of Darsigny et al. (2010). Short hairpin RNA sequences targeting the coding regions of *mcp*,* aer*,* cheV*,* cheB*,* cheW*,* cheV,* and *flgA* mRNAs were synthesized by Shanghai Generay Biotech Co., Ltd. (Shanghai, China) (Table S2). The annealed oligonucleotides were ligated using T4 DNA ligase (TaKaRa, Japan) to the pACYC184 vector double digested with *Bam*HI and *Sph*I. The recombinant plasmids were transformed into *E. coli* SM10 via heat shock and then transferred via conjugation from strain SM10 to ND‐01. An empty pACYC184 vector was used as the control. Chloramphenicol (34 μg/ml) was used to screen the stable silenced clones, which were then used for RNA extraction and in vitro adhesion, motility and capillary assays.

### 2.4 Transmission electron microscopy observation

Formvar‐coated grids were floated on 20‐μl drops of a bacterial cell suspension (Mantri et al., 2015). Excess sample was withdrawn by touching the edge of the grid to the cut edge of Whatman filter paper. The grids were negatively stained with a 1% solution of phosphotungstic acid and observed using a Tecnai F20 transmission electron microscope (Philips, Holland). More than 20 fields of view were randomly selected for each group. The flagellum lengths were measured from the images (*n* = 6 cells per condition) using Osiris 4.0 software (Geneva, Switzerland).

### 2.5 RNA extraction and reverse transcription

Total RNA from the bacteria was extracted using TRIzol (Invitrogen, Carlsbad, CA, USA) according to the manufacturer's recommended protocol (Bak, 2015). First‐strand cDNA was synthesized from 2 mg of total RNA using a Revert Aid Mu‐MLV cDNA synthesis kit (Invitrogen) according to the manufacturer's recommended protocol.

### 2.6 Quantitative real‐time PCR (QRT‐PCR)

The expression of differentially expressed genes (DEGs) belonging to the bacterial chemotaxis pathway was detected by qRT‐PCR using Power SYBR Green PCR Master Mix (Applied Biosystems, USA) according to the manufacturer's instructions (Remes, Eisenhardt, Srinivasan, & Klug, 2015). The expression levels were normalized using 16S RNA, and mean expression levels were calculated using the 2^−ΔΔCt^ method (*n* = 6). The primers are listed in Table S3.

### 2.7 Mucus preparation

This study was conducted in strict accordance with the recommendations outlined in the Guide for the Care and Use of Laboratory Animals of the National Institutes of Health. The protocol was approved by the Committee on the Ethics of Animal Experiments of the Animal Ethics Committee of Xiamen University (Acceptance No. XMULAC20120030).

Healthy large yellow croakers caught by commercial fishermen from marine cultured cages in the city of Ningde in Fujian Province, China, were used for mucus preparation according to our previously described method (Lin et al., 2017). After washing with sterile PBS (0.01 mol/L, pH 7.2), the skin mucus was harvested by scraping off the surface of the skin with a plastic spatula to remove the mucus gel layer. This layer was homogenized in PBS, and the homogenate was centrifuged twice (20,000*g*, 4°C, 30 min) to remove particulate materials and then filtered through 0.45‐ and 0.22‐μm pore size 200 filters. The mucus samples were adjusted to 1 mg protein/ml PBS using the method described by Bradford (1976).

### 2.8 In vitro adhesion assay

The bacterial adhesion assay was performed using the method described by Kong et al. (2015). A volume of 50 μl of mucus was evenly spread onto a 22‐mm × 22‐mm glass slide area and fixed with methanol for 20 min. Then, 1 ml of bacterial suspension (10^8^ CFU/ml) was placed on the mucus‐coated glass slides, incubated for 2 h at 25°C in a humidified chamber, and washed five times with PBS. Finally, the bacteria were fixed with 4% methanol for 30 min, dyed with crystal violet for 3 min, and counted under a microscope (×1,000). Three trials were conducted for each group, and 20 fields of view were selected. PBS instead of the bacterial suspension was used as the negative control.

### 2.9 Capillary assay

Bacterial chemotaxis was assessed with the capillary assay according to Zaval'skii, Marchenko, and Borovik (2003). A capillary tube with an inner diameter of 0.1 mm was sealed at one end and filled with mucus. The tube was dipped into a bacterial suspension containing ~10^8 ^CFU/ml. After incubation for 1 hr, the chemotaxis of bacterial cells was evaluated via comparison of the numbers of cells penetrating into the mucus‐containing tube and the numbers penetrating into the negative control tube, which was filled with buffer containing no mucus. The number of bacterial cells in the capillary tubes was accurately determined by plating the contents of the tubes onto agar medium. Three trials were conducted for each group.

### 2.10 Soft agar plate motility assay

The soft agar method was used for evaluating ND‐01 motility. Overnight cultures were diluted to an OD_660_ of 0.03. A 1‐μl drop of the cell suspension was spotted onto the center of LB plates (0.3% agar), and the cells were grown at 28°C for 20 hr, after which the diameters of the colonies were measured.

### 2.11 Data processing

The results are reported as means ± SD. The data were statistically analyzed with one‐way ANOVA followed by Dunnett's multiple comparison test using SPSS 13.0 software. A value of *p *<* *.05 was used to indicate a significant difference.

## 3 RESULTS

### 3.1 Effects of transient gene silencing

**Figure 1:**
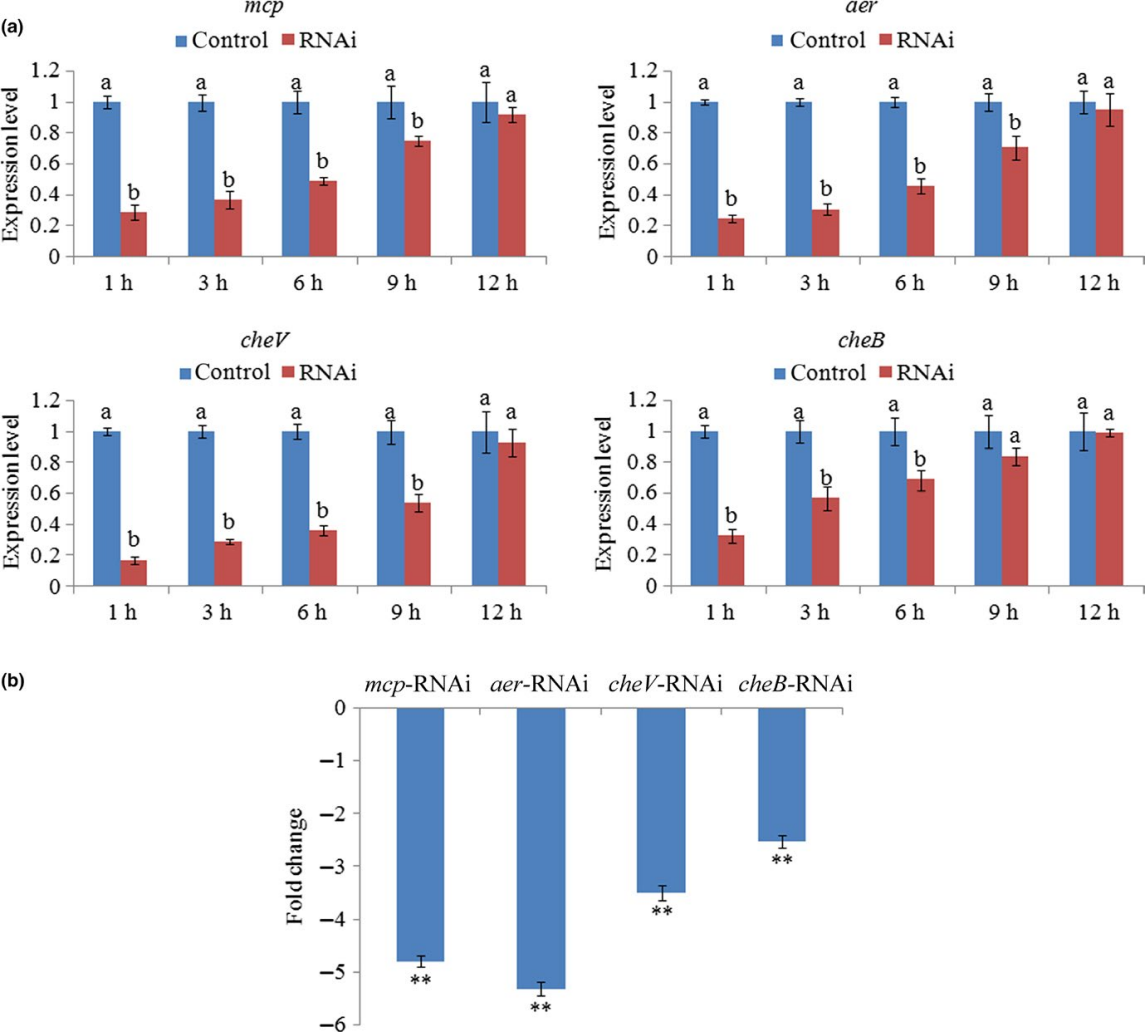
Transient gene silencing reduced ND‐01 adhesion. (a) qRT‐PCR analysis of *mcp*,* aer*,* cheV*, and *cheB* expression after transient gene silencing at 1, 3, 6, 9, and 12 h compared with the control. Data are presented as the means ± SD, and each treatment consisted of six independent biological replicates. The means of treatments not sharing a common letter are significantly different at *p *<* *.05. (b) Adhesion capacity of transiently silenced ND‐01 to mucus at 2 hr. Data are presented as the means ± SD Three independent biological replicates were included for each group. **p < .01 compared with the control

In order to evaluate the relationship between *mcp*,* aer*,* cheV*,* cheB,* and *V. alginolyticus* adhesion preliminarily, ND‐01 was treated with small interfering RNAs (siRNAs). Based on the percent decrease in expression (Figure 1a), ND‐01 treated with anti‐*mcp* siRNA, anti‐*aer* siRNA, and anti‐*cheV* siRNA showed significant (*p *<* *.05) gene silencing at 1–9 hr, whereas cells treated with anti‐*cheB* siRNA displayed significant gene silencing at 1–6 hr. Therefore, to validate the effects of transient gene silencing, the adhesion ability of *V. alginolyticus* under normal conditions and RNAi conditions was compared at 2 hr after electroporation. The results of the in vitro adhesion assay indicated significantly decreased ND‐01 adhesion ability under RNAi conditions (Figure 1b). After transient gene silencing for 2 hr, the adhesion ability of ND‐01 treated with *mcp‐* ,*aer‐*,* cheB‐,* and *cheV*‐RNAi was significantly reduced by 4.79‐, 5.31‐, 3.50‐, and 2.52‐fold.

### 3.2 Effects of stable gene silencing

**Figure 2:**
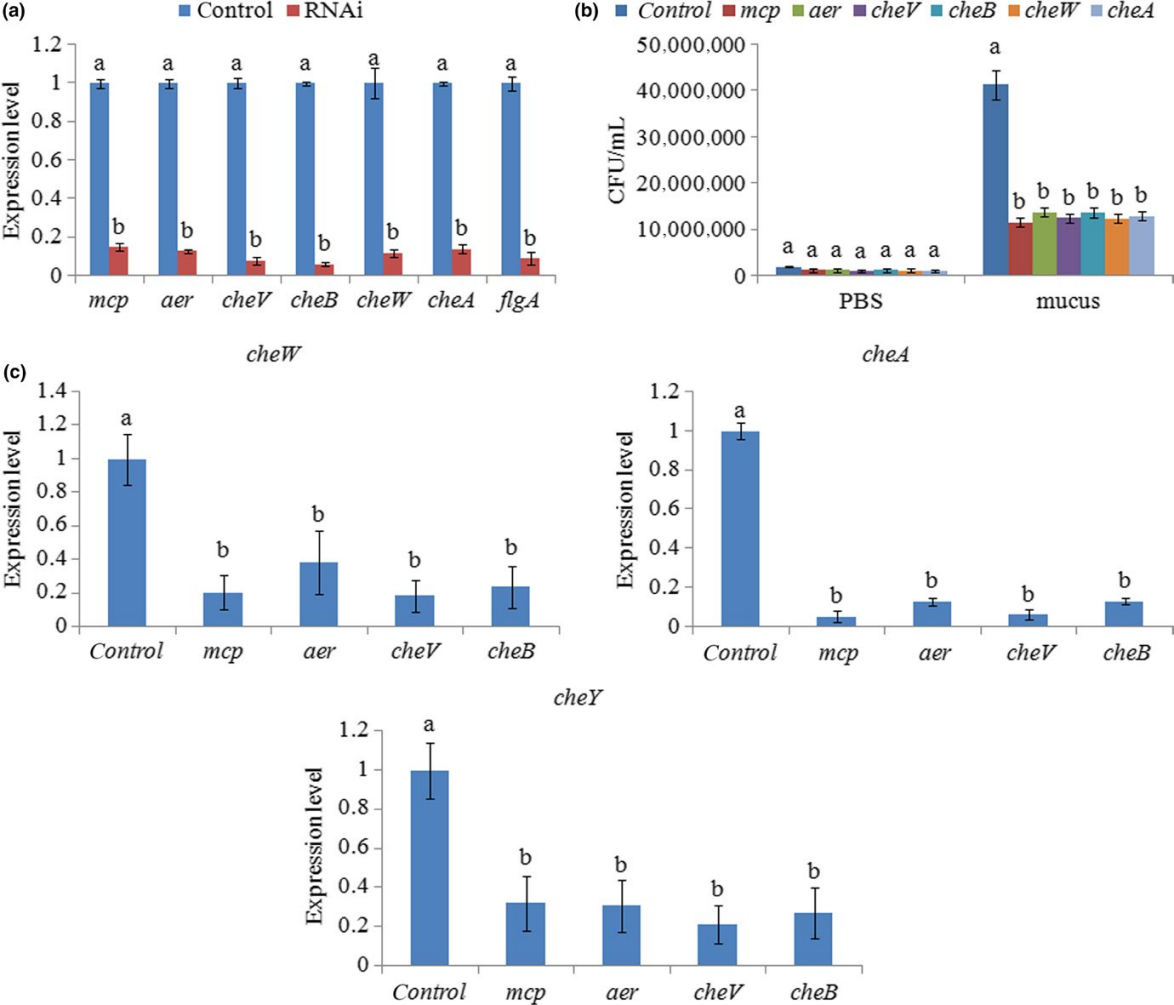
Stable gene silencing reduced the chemotactic ability of ND‐01. (a) qRT‐PCR analysis of *mcp*,* aer*,* cheV*,* cheB*,* cheW*,* cheA,* and *flgA* expression after stable gene silencing compared with the control. Data are presented as the means ±SD, and six independent biological replicates were performed per group. The means of treatments not sharing a common letter are significantly different at *p *<* *.05. (b) Chemotaxis capacity of stably silenced ND‐01 to mucus. Data are presented as the means ± SD. (*n* = 3). Means of treatments not sharing a common letter are significantly different at *p *<* *.05, as assessed using one‐way ANOVA followed by Dunnett's test. (c) qRT‐PCR analysis of *cheA*,* cheW,* and *cheY* expression after stable gene silencing compared with the control. Data are presented as the means ± SD, and six independent biological replicates were included for each group. The means of treatments not sharing a common letter are significantly different at *p *<* *.05

In order to further evaluate the role of *mcp*,* aer*,* cheV*, and *cheB*, stable gene silencing was carried out on ND‐01. As is shown in Figure 2a, the expression levels of *mcp*,* aer*,* cheV*, and *cheB* were significantly reduced by 6.67‐, 7.69‐, 12.50‐, and 16.67‐fold, respectively, in stably silenced clones. In addition, chemotactic ability was significantly reduced by stable gene silencing, with decreases of 3.56‐, 2.99‐, 3.30‐, and 3.00‐fold, respectively, for *mcp*,* aer*,* cheV*, and *cheB* (Figure 2b).

*cheA*,* cheY,* and *cheW* are three important downstream genes closely related to the functions of *mcp*,* aer*,* cheV*, and *cheB*, and their expression levels were detected in stably silenced ND‐01 (Figure 2c). Compared with the control group, the levels of *cheA*,* cheY,* and *cheW* expression were significantly reduced in all four RNAi groups. After stable gene silencing, *mcp* exhibited the smallest (by 6.67‐fold) decreases in expression, whereas after stable gene silencing of *mcp*, the expression of *cheA* presented the greatest (by 18.25‐fold) decreases. Therefore, the silencing of *mcp* had the greatest impact on *cheA*. After *cheB*‐RNAi, the expression of *cheA* decreased by 7.60‐fold, whereas after *aer*‐RNAi, the expression of *cheA* decreased by 7.59‐fold. Therefore, the decrease level of *cheA* after *cheB*‐RNAi and *aer*‐RNAi were very close. But, after stable gene silencing, the expression of *cheB* was decreased by 16.67 fold, whereas the expression of *aer* was decreased only by 7.69 fold. Taken together, the silencing of *cheB* had the lowest impact on *cheA*. For the same reason, compared to *aer*‐RNAi, *cheV*‐RNAi, and *cheB*‐RNAi, *mcp*‐RNAi appeared to have greater effect on *cheW*; whereas compared to *mcp*‐RNAi, *aer*‐RNAi, and *cheV*‐RNAi, *cheB*‐RNAi appeared to have greater effect on *cheY*.

**Figure 3:**
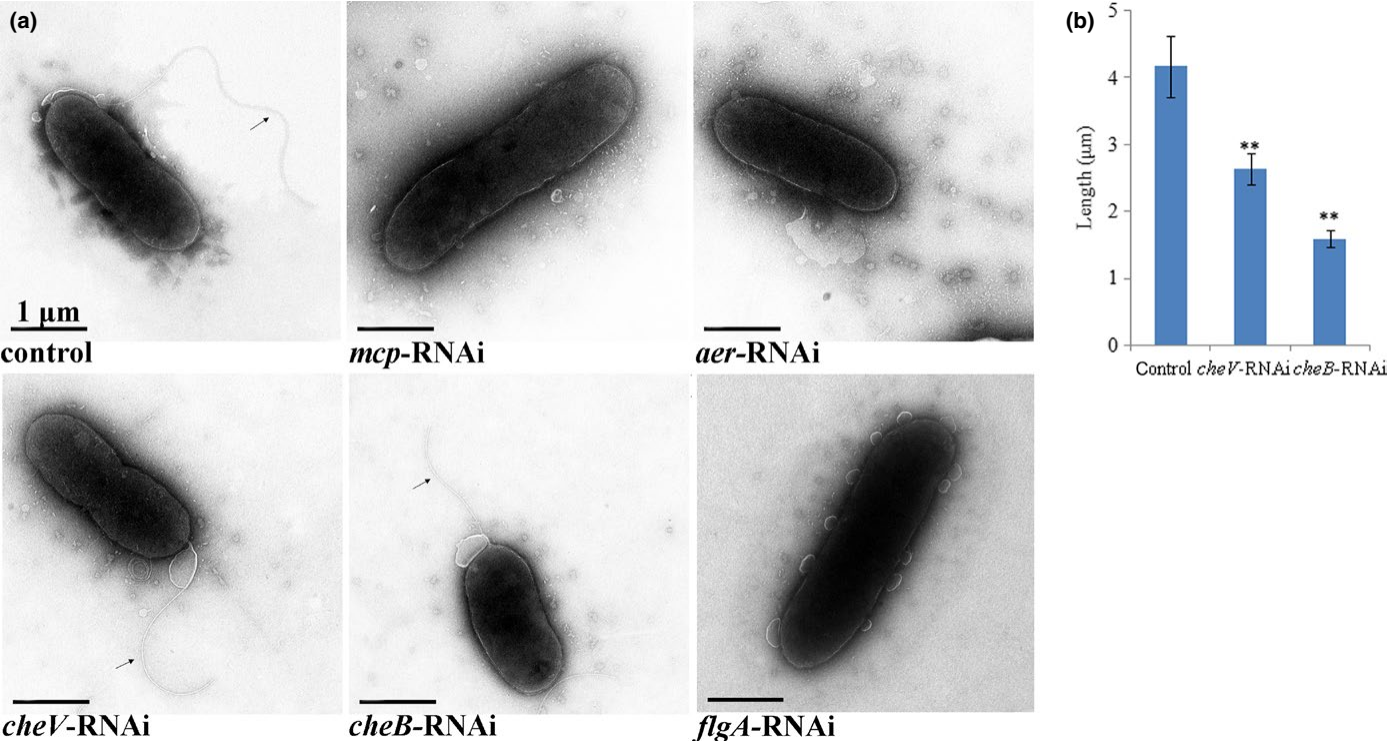
(a) Transmission electron micrographs of stably silenced ND‐01 cells. (b) Length of the flagella of control, *cheV*‐RNAi and *cheB*‐RNAi cells. Data are presented as the means ± SD; **p <* *.01 compared with the control

Following stable gene silencing, the flagella of ND‐01 were observed by transmission electron microscopy (Figure 3a). Although the observations revealed the presence of flagella on the cell surface of the control, *cheV*‐RNAi and *cheB*‐RNAi cells, the flagella of *cheV*‐RNAi and *cheB*‐RNAi cells were significantly shorter than those of the control (Figure 3b). No flagella were observed on the cell surface of the *mcp*‐RNAi and *aer*‐RNAi cells.

**Figure 4:**
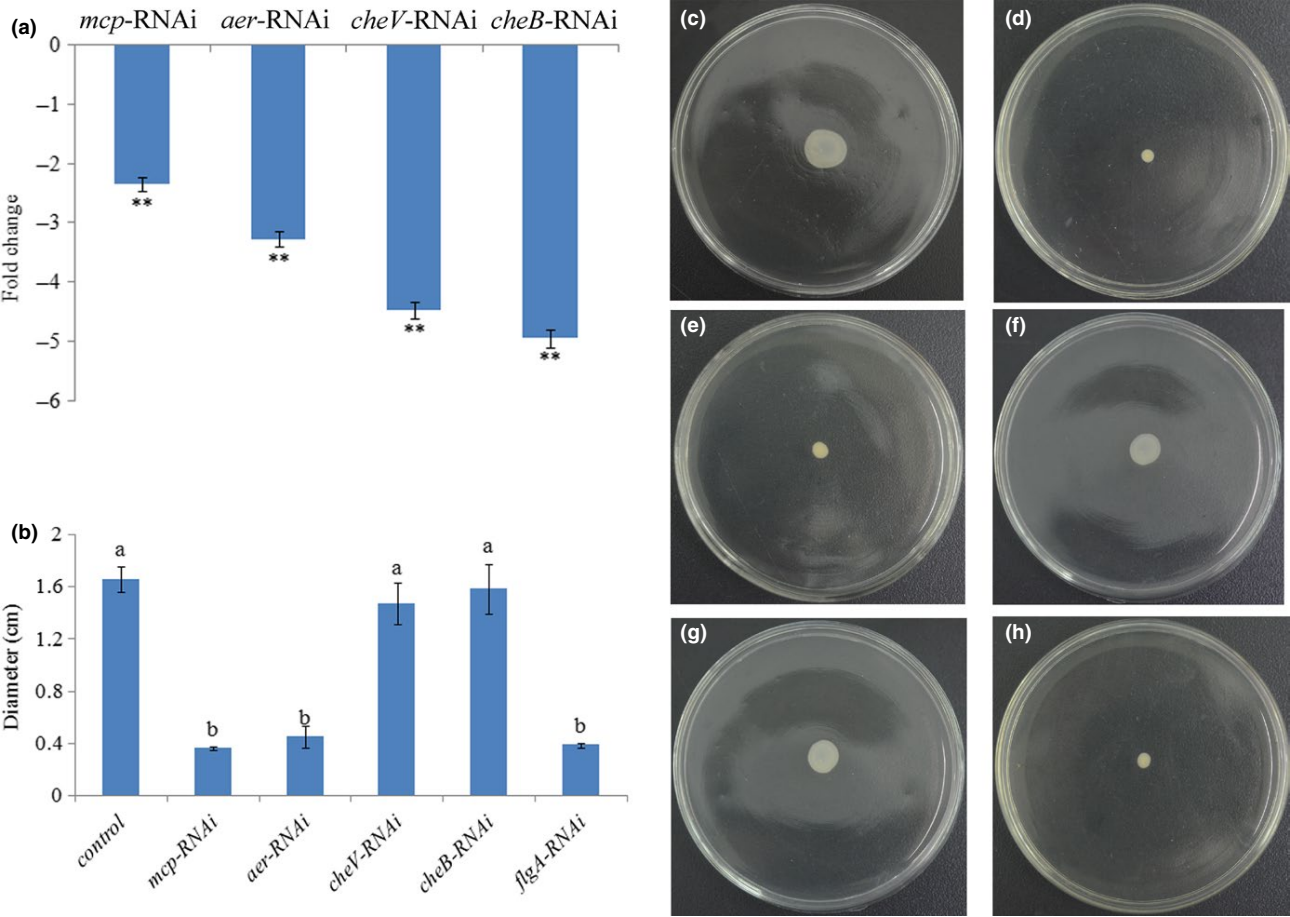
Stable gene silencing reduced the adhesion and motility of ND‐01. (a) Adhesion capacity of stably silenced ND‐01 to mucus. Data are presented as the means ± SD. Three independent biological replicates were included for each group. **p < .01 compared with the control. (b) Diameter of the colony of each strain. Data are presented as the means ± SD., and three independent biological replicates were included for each group. The means of treatments not sharing a common letter are significantly different at *p *<* *.05. (c–g) Typical images of spreading of control (c) and *mcp‐* (d), *aer‐* (e), *cheV‐* (f), *cheB‐* (g) and *flgA‐* (h) silenced ND‐01 cells

The adhesion ability of the stably silenced clones was assayed, which showed that the numbers of adherent bacteria of the control group were approximately 1,408 cells/view, whereas the corresponding numbers of adherent bacteria of the *mcp‐*,* aer‐*,* cheV‐*, and *cheB*‐RNAi groups were 598, 430, 315, and 284 cells/view, respectively (Figure 4a).

The motility of the stably silenced clones was also assessed, whereas no change was observed for *cheV*‐RNAi and *cheB*‐RNAi cells. Conversely, the motility of *mcp*‐RNAi and *aer*‐RNAi cells was significantly reduced (Figures 4b–g).

### 3.3 Effect of different temperatures

**Figure 5:**
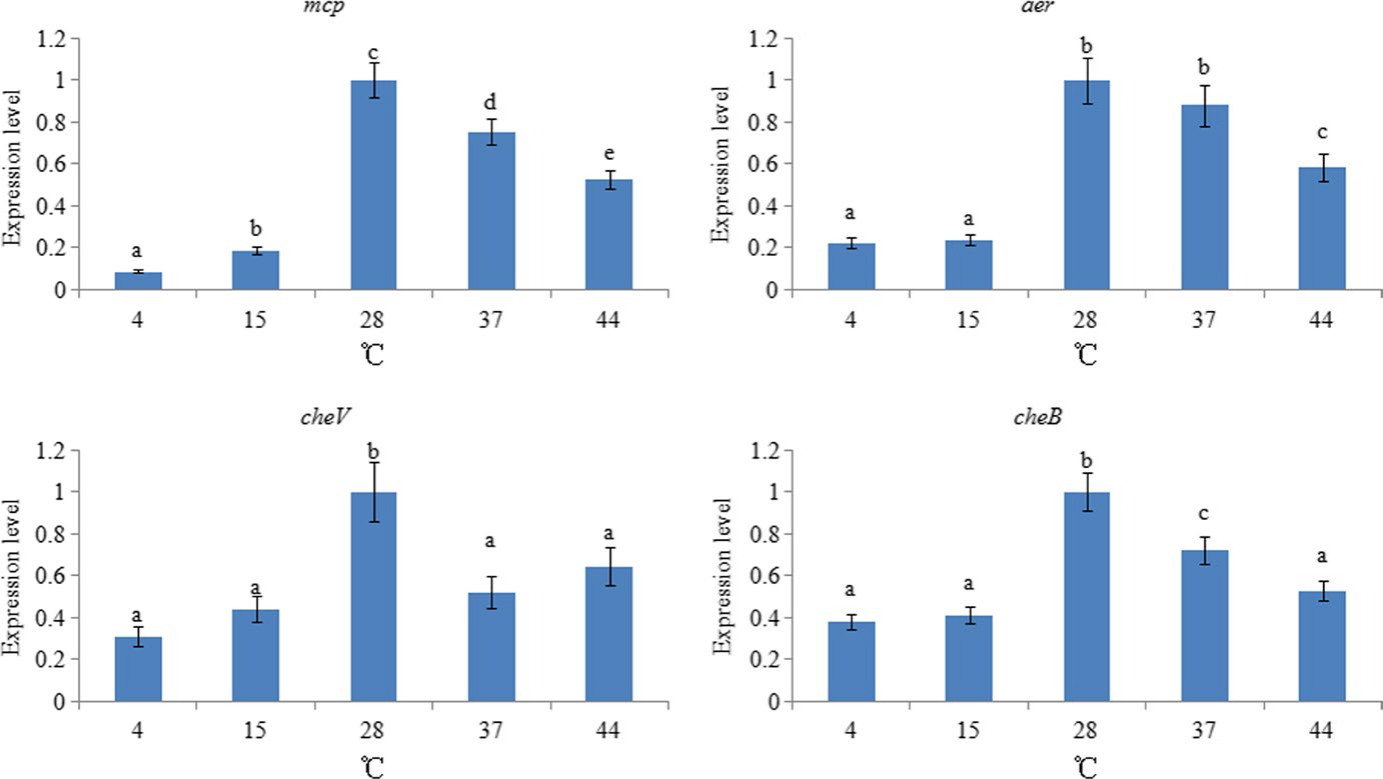
qRT‐PCR analysis of *mcp*,* aer*,* cheV*, and *cheB* expression in ND‐01 grown at different temperatures. Data are presented as the means ± SD, and each treatment consisted of six independent biological replicates. The means of treatments not sharing a common letter are significantly different at *p *<* *.05

In order to evaluate the response of these genes to temperature changes, the expression levels of the above genes were detected at different temperatures (Figure 5). *mcp*,* aer,* and *cheB* displayed a similar inverted U‐shaped trend, whereas the expression of *cheV* was not very regular. However, the highest expression of these genes was observed at 28°C. Based on the results, low temperatures apparently had a greater impact on *mcp*,* aer,* and *cheB* than high temperatures, and the *mcp* gene appeared to be the most sensitive to low temperature.

### 3.4 Effects of various pH treatments

**Figure 6:**
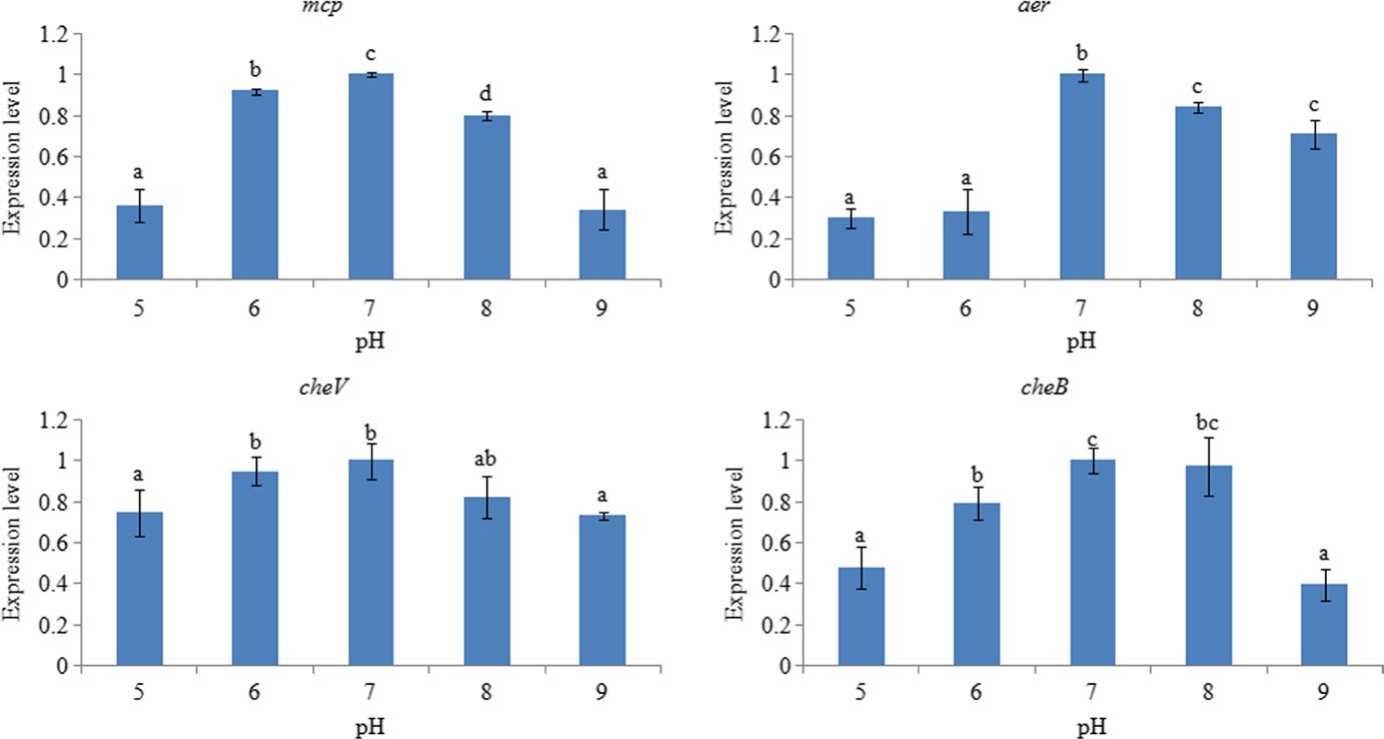
qRT‐PCR analysis of *mcp*,* aer*,* cheV*, and *cheB* expression in ND‐01 grown at various pH levels. Data are presented as the means ± SD, and each treatment consisted of six independent biological replicates. The means of treatments not sharing a common letter are significantly different at *p *<* *.05

In order to evaluate the response of these genes to pH changes, the expression of the four genes was also assessed under different pH levels (Figure 6), and again, a similar inverted *U*‐shaped trend was observed. The highest expression was observed at pH 7.0. The *aer* gene appeared to be the most sensitive to different pH, whereas *cheV* appeared to be the least sensitive.

### 3.5 Effects of different salinity treatments

**Figure 7:**
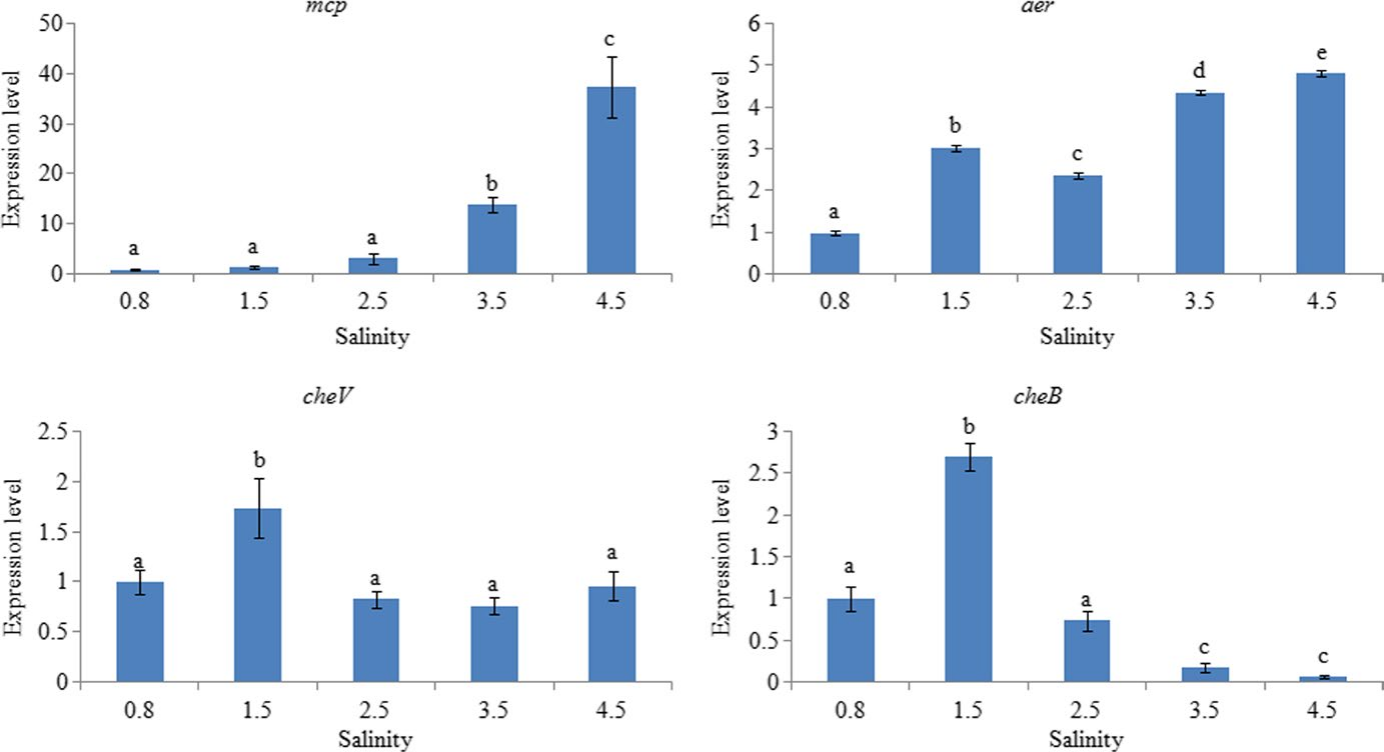
qRT‐PCR analysis of *mcp*,* aer*,* cheV*, and *cheB* expression in ND‐01 grown at different salinities. Data are presented as the means ± SD, and each treatment consisted of six independent biological replicates. The means of treatments not sharing a common letter are significantly different at *p *<* *.05

In order to evaluate the response of these genes to salinity changes, the effects of salinity on gene expression were detected and found to be quite different (Figure 7). When the salinity varied from 0.8% to 4.5%, the expression levels of *mcp* and *aer* were significantly increased in a salinity‐dependent manner; *cheB* expression displayed an inverted U‐shaped trend, reaching the highest levels at 1.5% salinity; *cheV* expression also reached the highest levels at 1.5% salinity, but the expression trend was not very regular. *mcp* displayed extreme sensitivity to low salinity, whereas *cheB* was rather sensitive to high salinity.

### 3.6 Effects of starvation

**Figure 8:**
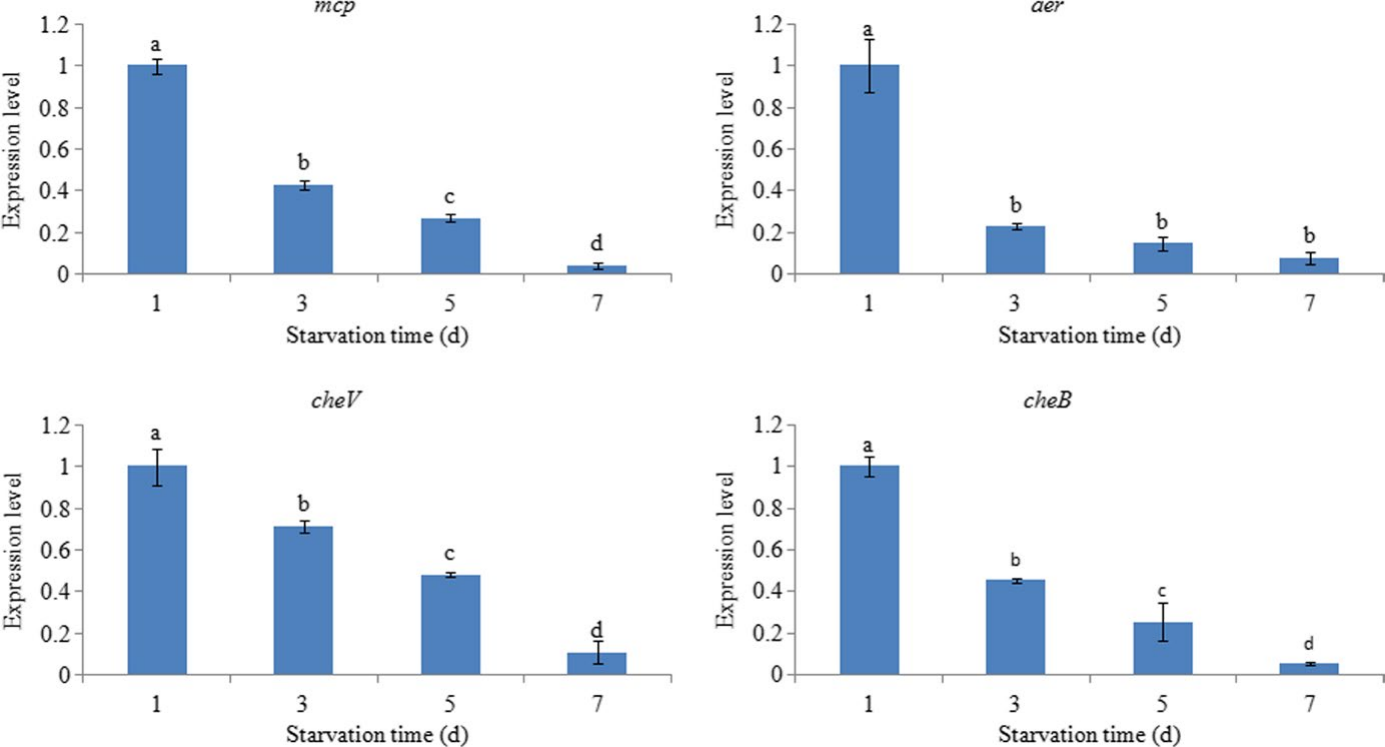
qRT‐PCR analysis of *mcp*,* aer*,* cheV*, and *cheB* expression in ND‐01 after different starvation times. Data are presented as the means ± SD, and each treatment consisted of six independent biological replicates. The means of treatments not sharing a common letter are significantly different at *p *<* *.05

In order to evaluate the response of these genes to starvation, the expression of the four genes was also assessed under starvation. Starvation significantly reduced gene expression in a time‐dependent manner (Figure 8). Furthermore, the expression of *aer* presented a sharp decrease after 3 days of starvation, whereas other three genes did not show such decreases, so it is more like that *aer* is most sensitive to starvation.

## 4 DISCUSSION

Zhang and Zhou (2013)sequenced three *Xanthomonas oryzae* pv. Oryzae stains using Illumina technology, and the results showed significant differences in expression of some genes related to chemotaxis and motility and that these differences affect virulence. This finding indicated that chemotaxis is not only closely related to virulence but that it is also tightly regulated by gene expression. Our RNA‐Seq results also revealed the relationship between the adhesion process of ND‐01 and the bacterial chemotaxis pathway. Furthermore, four commonly down‐regulated DEGs, *mcp*,* aer*,* cheV*, and *cheB* was identified, and it is probable that these four genes are the most sensitive to environmental conditions.

*mcp*,* aer*,* cheV*, and *cheB* are essential for bacterial chemotaxis. As transducers, the methylation status of MCPs determines the signal relayed to the flagellum, leading to movement toward an attractant or away from a repellent. In addition, a decrease in the number of MCPs transferring a signal to the flagellum slows bacterial motility (Parales et al., 2015). Aer is an aerotaxis, energy, and redox sensor (Bibikov et al., 2000; Samanta et al., 2016). Due to a strong bias in CW flagellar rotation (Cho et al., 2011), cheB_E_ mutants always tumble (Kanungpean, Kakuda, & Takai, 2011). Feedback by CheV inhibits signal transduction from receptors to the cytosol and thereby reduces chemotactic ability, which is consistent with gene silencing results. However, no previous study has focused on the transcriptional regulation of *mcp*,* aer*,* cheV*,* cheB,* and other genes of the bacterial chemotaxis pathway. According to our results, silencing of *mcp*,* aer*,* cheV*, and *cheB* affected the expression of other closely related downstream genes of the pathway, such as *cheA*,* cheW,* and *cheY*, which indicates that these four genes might regulate the expression of other genes and could have key roles in bacterial chemotaxis.

As shown by our results, *mcp*,* aer*,* cheV*, and *cheB* have a close relationship with adhesion, which is consistent with bioinformatic results and supports our hypothesis that the sensitivity of *mcp*,* aer*,* cheB,* and *cheV* to environmental stresses might constitute a mechanism through which environmental conditions influence adhesion. Interestingly, *mcp*‐RNAi and *aer*‐RNAi cells displayed decreased motility and flagellar assembly disorder. This finding is very similar to the results obtained in our previous research in which genes of the flagellar assembly pathway were silenced (Wang et al., 2015). As the bacterial chemotaxis pathway borders the flagella assembly pathway, the bacterial chemotaxis pathway might perturb flagellar assembly to affect motility and thereby regulate adhesion. However, stable silencing of *cheV* and *cheB* did not result in motility impairment. Therefore, motility is not the only approach through which the bacterial chemotaxis pathway influences adhesion, and further research is necessary for a full understanding of these processes.

Environmental factors, such as temperature, salinity, and pH, can markedly influence the bacterial adhesion capacity (Wang et al., 2015). As an important environmental factor, pH has significant influence on bacterial adhesion (Balebona et al., 1995; Yan et al., 2007), and heavy metals have also been shown to have effects on microorganisms (Haferburg & Kothe, 2007; Xiao, Zong, & Lu, 2015). Kong et al. (2015) showed that heavy metals, including Cu^2+^, Pb^2+^, and Hg^2+^, can significantly reduce ND‐01 adhesion to the skin mucus of large yellow croakers. Nonetheless, further details of the mechanism by which environmental factors regulate bacterial adhesion remain obscure. In this study, the effects of various environmental stresses, including temperature, pH, salinity and starvation, on the expression of *mcp*,* aer*,* cheV*, and *cheB* were examined. The four genes exhibited different responses under different environmental stresses, and the same environmental stress also led to different expression levels of the genes.

The change in adhesion ability at different temperatures displayed an inverted U‐shaped trend (Huang et al., 2016). The numbers of ND‐01 adhering to the skin mucus of large yellow croakers at 28°C was significantly higher than those observed at the other temperatures, which might be an explanation for the higher frequency of the disease caused by *V. alginolyticus* in early summer (Baker‐Austin, Stockley, Rangdale, & Martinez‐Urtaza, 2010; Morris & Black, 1985; Reilly, Reilly, Smith, & Baker‐Austin, 2011; Sterk, Schets, de Roda Husman, de Nijs, & Schijven, 2015). The trends observed by qRT‐PCR and the *in vitro* adhesion assay for different temperatures were quite similar, suggesting that temperature affects the adhesion of ND‐01 and that *mcp*,* aer*,* cheB,* and *cheV* might contribute to regulating adhesion at different temperatures. Furthermore, our results indicated that low temperatures had a greater impact on these genes than high temperatures, which might be due to the decreased metabolic level of ND‐01 at low temperatures.

ND‐01 adhesion capacity under different pH values displayed an inverted U‐shaped trend, reaching a peak at pH 7.0 (Huang et al., 2016). The trends obtained by qRT‐PCR and the in vitro adhesion assay for different pH levels were quite similar, indicating that pH affects ND‐01 adhesion and that *mcp*,* aer*,* cheB,* and *cheV* might contribute to regulating adhesion at different pH levels.

Maximal adhesion was achieved at a salinity of 3.5%. At 0.8% salinity, the level of adhesion was significantly higher than that detected at salinities of 1.5%, 2.5%, and 4.5% (Huang et al., 2016). The trends obtained by qRT‐PCR and the in vitro adhesion assay for different salinities were quite different and indicated that salinity affects ND‐01 adhesion and that the bacterial chemotaxis pathway might not be involved in the regulatory network governing adhesion under different salinities.

Our previous research showed no significant change in the number of culturable ND‐01 cells before 3 days of starvation (Huang et al., 2016); however, an increase in the duration of the treatment caused a substantial reduction in the number of bacteria adhering to skin mucus (Huang et al., 2016). Therefore, the decline in bacterial adhesion to skin mucus was mainly due to a decline in the bacterial adhesion ability rather than a decline in the number of bacteria in the suspension. These results suggested that vibriosis caused by ND‐01 was more likely to occur in eutrophic seawater than in oligotrophic seawater. Because the trends obtained by qRT‐PCR and the in vitro adhesion assay for starvation were quite similar, starvation does affect ND‐01 adhesion, and *mcp*,* aer*,* cheB,* and *cheV* might contribute to regulating adhesion under starvation conditions.

These findings indicate that the bacterial chemotaxis pathway plays a key role in the adhesion process of ND‐01 and is sensitive to certain environmental stresses, particularly pH and starvation. Because the pH of seawater is relatively stable (Roy & Tim, 2012), nutrients might be the main factor affecting bacterial chemotaxis and thus adhesion.

In conclusion, our results suggest the following: (1) *mcp*,* aer*,* cheV*, and *cheB* are closely associated with the process of adhesion in ND‐01; (2) *mcp*,* aer*,* cheV*, and *cheB* might affect adhesion by altering motility, though motility is not the only route through which adhesion is affected; and (3) *mcp*,* aer*,* cheV*, and *cheB* regulate adhesion in different natural environments. Together with our previous studies, environmental factor‐mediated regulation of adhesion might involve communication between the bacterial chemotaxis pathway and the flagellar assembly pathway, though further research is necessary to evaluate this possibility.

## CONFLICT OF INTEREST

None declared.
