# Supplementary material for: mcp, aer, cheB, and cheV contribute to the regulation of Vibrio alginolyticus (ND‐01) adhesion under gradients of environmental factors
Source: Microbiologyopen. 2017 Jul 25;6(6):e00517. doi: 10.1002/mbo3.517 (PMC5727358; doi:10.1002/mbo3.517)
Supplement: Supplementary file 1 [file MBO3-6-na-s001.doc]

**Table S1 siRNA Sequence**

| **Target gene** | **siRNA for transient gene silence** |
| --- | --- |
| *mcp* | F: 5' UAGAUCAAGCGACUCACGCTT 3'  R: 5' UAAAUAAUAGCGCUGUUGCTT 3' |
| *aer* | F: 5' GCGUGAGUCGCUUGAUCUATT 3'  R: 5' UAGAUCAAGCGACUCACGCTT 3' |
| *cheV* | F: 5' GCACUUAGUGAAGAGACUATT 3'  R: 5' UAGUCUCUUCACUAAGUGCTT 3' |
| *cheB* | F: 5' GCAGGAAUAUCUCACCCAUTT 3'  R: 5' AUGGGUGAGAUAUUCCUGCTT 3' |
| Negative control | F: 5'-UUCUCCGAACGUGUCACGUTT-3'  R: 5'-ACGUGACACGUUCGGAGAATT-3' |
