# Supplementary material for: mcp, aer, cheB, and cheV contribute to the regulation of Vibrio alginolyticus (ND‐01) adhesion under gradients of environmental factors
Source: Microbiologyopen. 2017 Jul 25;6(6):e00517. doi: 10.1002/mbo3.517 (PMC5727358; doi:10.1002/mbo3.517)
Supplement: Supplementary file 2 [file MBO3-6-na-s002.doc]

**Table S2 Oligonucle**otides used in producing shRNA for stable gene silencing

| **Target gene shRNA sequence for** **stable gene silence** | |
| --- | --- |
| *mcp* | F:5'-GATCCTAGATCAAGCGACTCACGCTTTTCAAGAGAAAGCGTGAGTCGCTTGATCTATTTTTTGCATG-3'  R:5'-CAAAAAATAGATCAAGCGACTCACGCTTTCTCTTGAAAAGCGTGAGTCGCTTGATCTAG-3' |
| *aer* | F:5'-GATCCGCGTGAGTCGCTTGATCTATTTTCAAGAGAAATAGATCAAGCGACTCACGCTTTTTTGCATG-3'  R:5'-CAAAAAAGCGTGAGTCGCTTGATCTATTTCTCTTGAAAATAGATCAAGCGACTCACGCG-3' |
| *cheV* | F:5'-GATCCGCACTTAGTGAAGAGACTATTTTCAAGAGAAATAGTCTCTTCACTAAGTGCTTTTTTGCATG-3'  R:5'-CAAAAAAGCACTTAGTGAAGAGACTATTTCTCTTGAAAATAGTCTCTTCACTAAGTGCG-3' |
| *cheB* | F:5'-GATCCGCAGGAATATCTCACCCATTTTTCAAGAGAAAATGGGTGAGATATTCCTGCTTTTTTGCATG-3'  R:5'-CAAAAAAGCAGGAATATCTCACCCATTTTCTCTTGAAAAATGGGTGAGATATTCCTGCG-3' |
