# Supplementary material for: mcp, aer, cheB, and cheV contribute to the regulation of Vibrio alginolyticus (ND‐01) adhesion under gradients of environmental factors
Source: Microbiologyopen. 2017 Jul 25;6(6):e00517. doi: 10.1002/mbo3.517 (PMC5727358; doi:10.1002/mbo3.517)
Supplement: Supplementary file 3 [file MBO3-6-na-s003.doc]

**Table S3 Primers for q RT-PCR**

| **Gene** | **Primers for qRT-PCR** |
| --- | --- |
| *mcp* | F: 5' GGTTGAGGGCTCTTCTGTGGTC 3'  R: 5' TGTACGAACTTCATCAGCAACGAC 3' |
| *aer* | F: 5' TGAACAAATCCAAGCGTCTATCTCT 3'  R: 5' GCTCTACGGAGTGCGTTGCT 3' |
| *cheV* | F: 5' TCATCGCGGATATTAACCCA 3'  R: 5' GATCACATTGTAACCTGAAGAAGCT 3' |
| *cheB* | F: 5' GGCAGGCTGGACGAACTATC 3'  R: 5' GAACTTGGCAATGCTTGTGGA 3' |
| *cheA* | F: 5' CCTTGATGTGGTAACTGCGG 3'  R: 5' GGAACTACACCATTGACGCC 3' |
| *cheW* | F: 5' GAGTTAGGTGGTATTCATCGCAT 3'  R: 5' CTCAATCCACCATAAGTAGCGTA 3' |
| *cheY* | F: 5' ATTTCCGATCATGTCATGCCA 3'  R: 5' TAAAGGCTAGTACAGTACGGT 3' |
| *16S RNA* | F: 5'-GGGGAGTACGGTCGCAAGAT-3'  R: 5'-CGCTGGCAAACAAGGATAAGG-3' |
